# SOLITUDE LINKS WITH INDIVIDUAL AND RELATIONAL WELL-BEING: EVIDENCE FROM DYADIC DAILY LIFE ASSESSMENTS

**DOI:** 10.1093/geroni/igad104.1059

**Published:** 2023-12-21

**Authors:** Elizabeth Zambrano Garza, Rachel Murphy, Maureen Ashe, Kenneth Madden, Denis Gerstorf, Christiane Hoppmann, Theresa Pauly

**Affiliations:** University of British Columbia, Vancouver, British Columbia, Canada; University of British Columbia, Vancouver, British Columbia, Canada; University of British Columbia, Vancouver, British Columbia, Canada; University of British Columbia, Vancouver, British Columbia, Canada; Humboldt University of Berlin, Berlin, Berlin, Germany; University of British Columbia, Vancouver, British Columbia, Canada; Simon Fraser University, Vancouver, British Columbia, Canada

## Abstract

Solitude, defined as physical aloneness or the absence of social interactions, has been associated with both positive and negative outcomes. Older adults spend a significant amount of their waking time by themselves (exacerbated during the pandemic), but they may be better equipped to regulate the negative emotions that are often linked with being alone. Additionally, solitude may provide a balance between social connectedness and autonomous needs. Using pandemic, end-of-day daily diary data across 10 days from 136 older Canadian adults and a close other of their choice (59% spouses, M = 66.49 years, SD = 13.26 range: 18-87 years, 88% White, 62% women), this project aims to examine associations of actor and partner solitude with relationship quality and daily affect. Multilevel models revealed that participants who reported more voluntary solitude experienced more daily positive affect and those who reported more negative solitude experienced both more negative affect and less positive affect at both daily and overall levels. We also found daily partner effects such that more voluntary solitude of the partner was associated with less actor negative affect and negative solitude of the partner being associated with less actor positive affect and more negative affect over and above actor effects. Finally, when partners reported more voluntary solitude, participants reported higher relationship support. Findings shed light on the benefits and drawbacks of how solitude is experienced in older adulthood, highlighting the influence social partners affect quality and relationship satisfaction.

